# Different lead locations guided by fluoroscopy and ECG parameters and their effect on patients functional status

**DOI:** 10.1186/s43044-019-0023-1

**Published:** 2019-11-28

**Authors:** Mohamed MesbahTahaHassanin, Ahmad ShafieAmmar, Radwa M. Abdullah, Mohammad Hassan Khedr

**Affiliations:** Cardiology Department, Faculty of Medicine, Zagazig University, Villa 7, District 11/12, 1st Settlement, New Cairo, Zagazig, Egypt

**Keywords:** Pacing, Different lead locations, QRS vector and duration, Fluoroscopy, 6 MWT

## Abstract

**Background:**

Right ventricular apical pacing with the resultant left ventricular dyssynchrony often leads to depressed systolic function and heart failure. This study aimed at investigating the relation between various septal locations guided by ECG and fluoroscopy and the intermediate term functional capacity of the patients.

**Results:**

Fifty patients who received a single lead pacemaker with assumed > 90% pacemaker dependency. Patients were randomized according to RV pacing site RV into group 1 “high septum” (*n* = 15), group 2 “mid septum” (*n* = 25), and group 3 “low septum” (*n* = 10) using QRS vector and duration as well as fluoroscopic parameters. Their clinical status was assessed 6 months after device implementation using 6-min walk test (6MWT).

The study showed that paced QRS complex duration itself has no significant difference between the different septal pacing locations (*P*-value 0.675), although its combination with the paced QRS complex vector can signify the optimal pacing site and 6MWT showed a significant difference among the groups in favor of group 1; group 1 (413.3 ± 148.5), group 2 (359.8 ± 124.6), and group 3 (276.0 ± 98.5) *P* value 0.04.

**Conclusion:**

There was a significant difference found between the three septal pacing sites concerning the patient functional capacity with superiority of high septal location. By contrast, different septal sites showed no significant difference of the paced QRS complex duration. To optimize the pacing site in the septum, assessment of the paced QRS vector in leads I and III is of a great benefit especially when combined with paced QRS complex duration assessment.

## Background

Right ventricular (RV) apical pacing allows for safe and stable long-term pacing. However, it induces non-physiologic left ventricular (LV) activation with alteration of the intraventricular contraction sequence, which delays LV activation [1]. This delay is accompanied with LV dyssynchrony, and the development of LV dyssynchrony was reported to be associated with deterioration of heart failure symptoms and the systolic LV dysfunction [2].

To overcome this potential limitation, other pacing sites throughout the whole septal segments have been tried [3]. The septal areas, particularly the mid-right ventricular (RV) septum and the RV outflow tract (RVOT), have been proposed as alternative pacing sites to RV apical pacing leading to a more physiologic electrical conduction to the left ventricle (LV) and therefore to a more physiologic contraction [4].

The degree of electrical dyssynchrony induced by RV pacing can be estimated by the duration of the paced QRS. Moreover, some studies have demonstrated that patients with a long paced QRS may be at high risk of adverse cardiac events including heart failure. A long paced QRS can result despite the non-apical placement because the lead might pace a non-favorable septal segment [5].

The aim of our study is to investigate the relation between various septal locations guided by ECG and fluoroscopy and the intermediate term functional capacity of the patients through illustrating the relationship between different septal positions and paced QRS complex duration and the relationship between the paced QRS vector and duration of the paced QRS complex.

## Methods

This cross-sectional study conducted during the period from October 2017 to July 2019 at our cardiology department on 50 patients having a primary implant single lead VVI pacemaker following the recent guidelines for cardiac pacing with high probability of device dependency (more than 90%) excluding those with significant valve disease (i.e., mitral insufficiency 75% and worse, moderate or severe aortic stenosis), recent (within 3 months) acute coronary syndrome, planned cardiac surgery (coronary artery bypass grafting, valve surgery), and ejection fraction of left ventricle less than 50%.

A detailed informed verbal consent has been taken from all participants as it was just an observational study and this consent was approved by our ethics committee.

Fifty patients were divided into three groups depending on the septal location with fluoroscopy guidance: group 1 high septum, group 2 mid septum, and group 3 low septum.

All participants were subjected to a follow up visits for 6 months after implantation of the device. A detailed history taking, physical examination, ECG data (including assessment of paced QRS complex duration and vector configuration), and assessment of their functional status using 6MWT were done in our cardiology department.

### Fluoroscopic definition of septal pacing sites

**Fig. 1:**
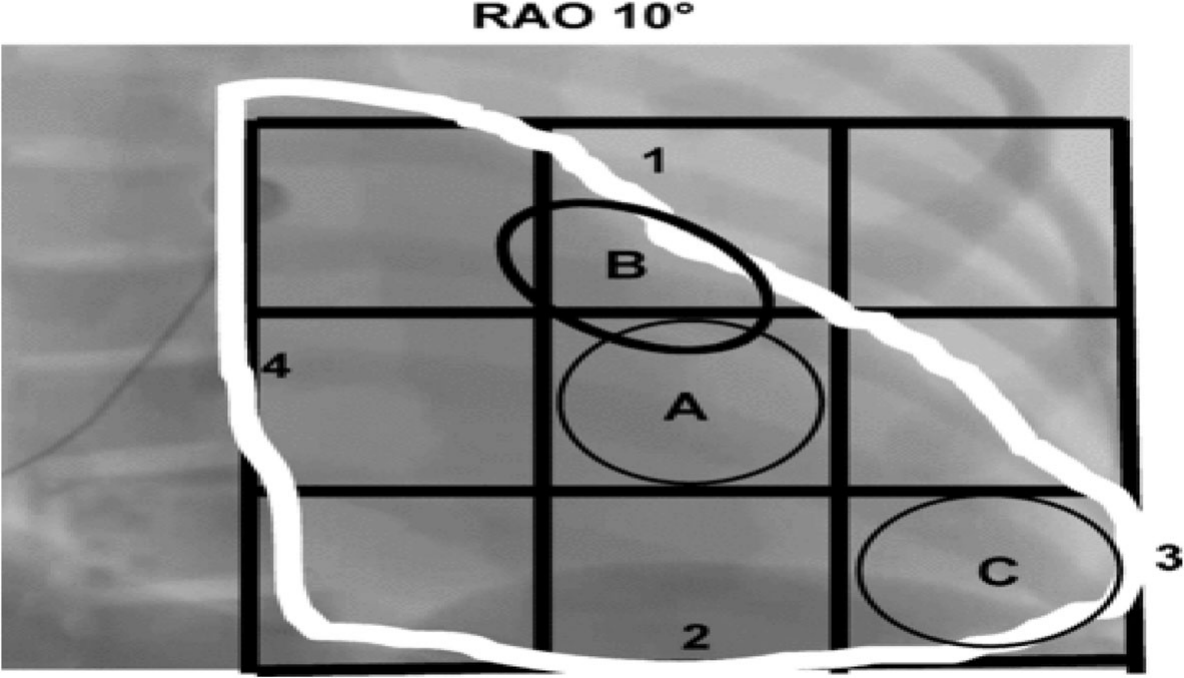
RAO view circles A and B show the approximate location of the septal lead in optimize RV and protect pace, respectively. Circle C shows the approximate location of the RV apical lead

**Fig. 2:**
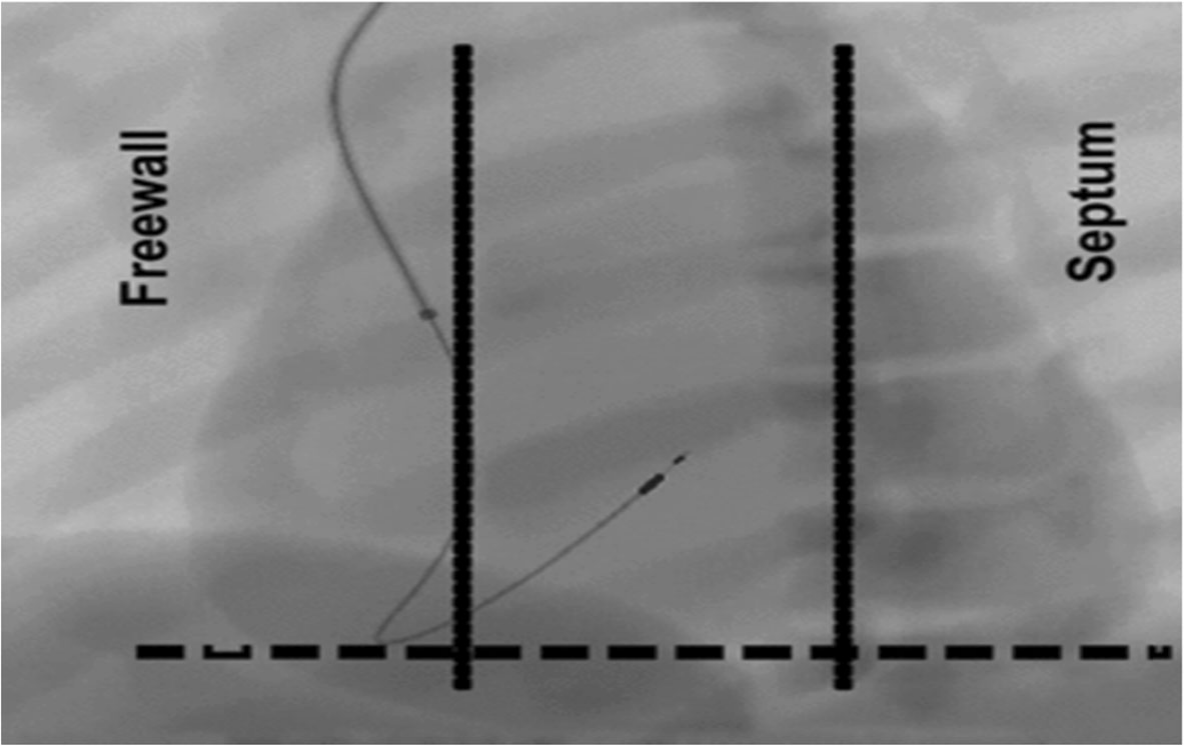
LAO 40 view to differentiate lead placement on the RV septum from the RV free wall

To facilitate lead placement in the RV septum, a 3 × 3 square grid is created for each patient using a fluoroscopic image in the right anterior oblique (RAO) 10° view using landmarks to demarcate the borders of the grid as shown in (Fig. 1) that facilitate to clarify the high, mid, and low septal locations. After fixation of the septal lead using the RAO view, the left anterior oblique (LAO) view (40–45°) is used to confirm that the lead is on the septum and not the free wall (Fig. 2) [6]

### Determining the paced QRS complex duration

All ECG records are made using an identical ECG device (Nihon Kohden Cardiofax C ECG-1150. Japan) of limb leads I, II, and III at speed 50 mm/s at a ventricular rate less than 80 bpm. The paced QRS complex duration is calculated from the end of the pacing spike to the latest deflection of the QRS complex in any limb lead. The non-paced QRS complex duration is measured from the earliest deflection to the latest deflection of the QRS complex in any limb leads. Optimization of the pacing site of the RV septum occurred by the maximal shortening of the QRS complex duration.

### Defining the paced QRS complex vector

Assessment of the paced QRS complex vector is simplified to determine the R/S voltage ratio in leads I and III. Three categories are defined positive, isoelectric, and negative. A positive vector is determined as R-S ratio > 1/2 R. An isoelectric vector is determined as R-S ratio < 1/2 R or S-R ratio < 1/2 S. A negative vector is defined as S-R ratio > 1/2 S. Then, they are divided into three subgroups: (1) negative or isoelectric in lead I plus positive in lead III. (2) Isoelectric or negative in lead III. (3) Positive in lead I plus positive in lead III [7].

### Statistical analysis

Standard descriptive statistics were used for the analysis; continuous parameters of QRS duration were described by means and standard deviation, while occurrences of categorical parameters (gender, pacing site) were described by count and percentages.

Differences between parameters of QRS duration and vector according to several factors and their combination were assessed by ANOVA and also by chi-square tests. For the studied groups, statistically significant differences at the level of significance of 0.05 are stated.

Post hoc tests analysis was done over the paced QRS complex vector result and also the 6MWT result to compare each group with the other group specifically.

For data analysis, the SPSS Statistics 17.0 for Windows (SPSS Inc., Chicago, IL, USA) was used and α = 0.05 was considered as the level of statistical significance in all analyses.

## Results

Fifty patients were divided according to the RV lead pacing site into group 1 “high septum” (*n* = 15), group 2 “mid septum” (*n* = 25), and group 3 “low septum” (*n* = 10). There are three patients who were excluded through the follow-up period and the reasons were death, ischemic cerebral stroke, and non-compliance. No other complications have been reported.

The demographic characteristics of the 50 patients are shown in (Table 1). Risk factors were adjusted in the three groups to eliminate interference of risk factors on the results and revealed no significant difference between the groups.

**Table 1:** Patients demographics

	Group 1 (*n* = 15)	Group 2 (*n* = 25)	Group 3 (*n* = 10)	*P* value
Age	63.5 ± 6.6	64.7 ± 7.5	68.8 ± 11.4	0.275
HTN	13 (86%)	16 (64%)	7 (70%)	0.299
DM	5 (33%)	11 (44%)	4 (40%)	0.801
Smoking	7 (46%)	11 (44%)	3 (30%)	0.682
Dyslipidemia	3 (20%)	4 (16%)	2 (20%)	0.934
Female gender	6 (40%)	6 (24%)	4 (40%)	0.479

Demographic risk factors revealed no significant difference between the groups.

All patients were subjected to vector configuration using leads I and III and classified into to three configurations accordingly: A “negative or isoelectric in lead I and positive in lead III” (*n* = 19), B “isoelectric or negative in lead III” (*n* = 15), and C “positive in lead I and positive in lead III” (*n* = 16).

Patients’ clinical status was assessed after VVI pacemaker implementation within the follow-up period using 6-min walk test (6MWT) counting the meters the patient can walk freely through the examination time and taking its all precautions in consideration.

## Discussion

The septal position of the pacing lead in the right ventricle is a preferred alternative pacing site given the potential contribution of limiting LV dyssynchrony compared to RV apex stimulation. Historically, the decision of where in the septum the lead should be placed is individual, with the selection being corrected according to the paced QRS complex duration [7].

In our study, 50 patients were divided according to the RV lead pacing site by new documented fluoroscopic method into group 1 “high septum” (*n* = 15), group 2 “mid septum” (*n* = 25), and group 3 “low septum” (*n* = 10), all of them were subjected to follow-up for 6 months after VVI pacemaker implantation.

Risk factors were adjusted in the three groups to eliminate interference of risk factors on the results (Age, gender, smoking, HTN, DM, and dyslipidemia) with no significant difference between the groups.

According to the functional capacity assessment, there is significant difference that was found between the three septal pacing site (high, mid, and low) with significant superiority of high septal location.

By contrast, different septal sites defined by the mentioned fluoroscopic method in the RAO 10° view showed no significant differences in paced QRS complex duration. But, assessment of the paced QRS vector in leads I and III is of a great benefit especially when combined with paced QRS complex duration assessment as group C of the QRS vector configuration showed significant longest QRS duration.

According to the paced QRS complex duration, there is no significant difference among the groups concerning the paced QRS duration, which was in agreement with [7] whose RCT evaluated paced QRS duration in various septal positions in a total of 609 patients where the RV lead was allocated using fluoroscopic guidance and stressed on the superiority of QRS vector configuration above the QRS duration in septal lead insertion.

And we disagree with another randomized controlled trial by [8] showed that RVOT septal pacing is associated with shorter QRS duration than elsewhere in the RV outside the His bundle. This suggests that pacing from the septal RVOT, although not as good as intrinsic conduction, may be the most desirable site for chronic RV pacing as a narrow QRS duration is associated with improved LV dynamics.

Another multi-center prospective study comparing the ventricular dyssynchrony according to the position of right ventricular pacing electrode [11] where pacemaker leads were inserted through the subclavian vein using standard implantation techniques. The RV leads were positioned in the RV apex (*n* = 45) or interventricular septum (*n* = 34) under fluoroscopic guidance. The results showed that the QRS duration was significantly increased in both groups after pacing, but the difference between the pre- and post-pacing QRS duration was significantly higher in apical pacing group (57.1 ± 28.3 versus 32.8 ± 40.5 ms). This study concluded that the apical pacing has a higher probability of more LV dyssynchrony after PM implantation.

Regarding the QRS vector configuration our study show high statistical significant difference among the groups in favor of group I high septum which goes with the result of a meta-analysis by ([8, 9] which both concluded that high septum and RVOT have been suggested as more physiological sites for cardiac Pacing and the ECG confirmation of pacing in the right ventricular septum is manifested by a negative QRS morphology in lead I, whereas pacing in the right ventricular free wall manifests as a positive QRS morphology in lead I. Also, ventricular pacing in a high position will result in an upright QRS in aVF, whereas a lower position will have a less positive QRS deflection in aVF.

A prospective observational study studied the optimal pacing site in the right ventricular septum [10]. Overall, 304 measurements of paced QRS complex characteristics in different RVS sites were performed in (100/102 patients). This study concluded that leads I and II together with fluoroscopy view can provide useful information for selecting the optimal pacing site corresponding to the QRS duration shorter than 160 ms as a cut-off value for HF prediction (Tables 2, 3, 4, and 5).

**Table 2:** Differences in paced QRS duration according to septal pacing site in the three groups

	Paced QRS duration (ms) Mean ± SD	*P* value
Group 1	143.7 ± 10.4	0.675
Group 2	143.9 ± 12.8	
Group 3	147.7 ± 13.15	

There is no significant difference found between the three groups of lead pacing site concerning the paced QRS duration

**Table 3:** Comparison of paced QRS groups regarding vector in leads I and III toward the groups of pacing site

	Paced QRS vector configuration			*P* value
	A (*n*)	B (*n*)	C (*n*)	
Group 1	8 (53%)	4 (27%)	3 (20%)	0.045
Group 2	9 (36%)	10 (40%)	6 (24%)	0.057
Group 3	2 (20%)	1 (10%)	7 (70%)	0.02

Group 2 “mid septum” has no significant difference regarding the QRS vector configuration, but group 1 “high septum” has significant difference that 53% of them has QRS vector configuration as group A and group 3 “low septum” has significant difference that 70% of them has QRS vector configuration as group C

**Table 4:** Comparison of paced QRS duration difference as regards the paced QRS vector groups in leads I and III

Vector configuration	Paced QRS duration (ms)			*P* value
	Mean ± SD			
A	136.5	9.4		0.001
B	142.2	7.9		
C	156.4	8.6		
Paced QRS vector groups “I”	“J”	Paced QRS duration (m)		*P* value
		Mean difference “I–J”	Std error	
Group A	B C	− 5.74035 − 19.91118*	3.02522 2.97192	0.064 0.001
Group B	A C	5.74035 − 14.17083*	3.02522 3.14786	0.064 0.001
Group C	A B	19.91118* 14.17083*	2.97192 3.14786	0.001 0.001

According to the paced QRS vector groups and the QRS duration, there is no significant difference between groups A and B but there is markedly high significant difference between A and C and also between B and C which illuminates the result that groups A and B have significant shorter QRS duration than group C

**Table 5:** Clinical functional capacity assessment through 6MWT results

		6MWT in meters Mean ± SD		*P* value
Group 1		413.3 ± 148.5		0.04*
Group 2		359.8 ± 124.6		
Group 3		276.0 ± 98.5		
		6MWT (meters)		*P* value
		Mean difference	SE	
Group 1	2	53.453	41.780	0.207
	3	137.333*	52.225	0.012
Group 2	1	− 53.453	41.780	0.207
	3	83.880	47.865	0.086
Group 3	1	− 137.333*	52.225	0.012
	2	− 83.880	47.865	0.086

According to the functional capacity clinical assessment, there is a significant difference that was found between the three groups confirmed by 6MWT

*The difference is highly significant between group 1 and group 3 in favor of group 1 (high septal location)

## Conclusion

In a total of 50 patients, there is significant difference was found between the three septal pacing site (high, mid, and low) concerning the patient functional capacity with superiority of high septal location.

By contrast, different septal sites defined by the mentioned fluoroscopic criteria in the RAO 10° view showed no significant difference of paced QRS complex duration.

To optimize the pacing site in the septum, assessment of the paced QRS vector in leads I and III is of a great benefit especially when combined with paced QRS complex duration as when the vector configuration was “positive in lead I and positive in lead III,” it showed significant longest QRS duration.

## Data Availability

Available in my institute with a case rate of 3–4 cases per month. Available in Cardiology Department, Faculty of Medicine, Zagazig University, Zagazig, Egypt, with a case rate of 3–4 cases per month.

## Abbreviations

6MWT: Six-minute walk test
DM: Diabetes mellitus
ECG: Electrocardiography
HF: Heart failure
HTN: Hypertension
LV: Left ventricular
PM: Pacemaker
RCT: Randomized control trial
RV: Right ventricular
RVOT: Right ventricular outflow tract
SD: Standard deviation
